# The Effect of Monochromatic LED Light Wavelengths and Photoperiods on *Botrytis cinerea*

**DOI:** 10.3390/jof7110970

**Published:** 2021-11-16

**Authors:** Neringa Rasiukevičiūtė, Aušra Brazaitytė, Viktorija Vaštakaitė-Kairienė, Asta Kupčinskienė, Pavelas Duchovskis, Giedrė Samuolienė, Alma Valiuškaitė

**Affiliations:** 1 Laboratory of Plant Protection, Institute of Horticulture, Lithuanian Research Centre for Agriculture and Forestry, Kauno St. 30, LT-54333 Babtai, Kaunas Dist., Lithuania; alma.valiuskaite@lammc.lt; 2 Laboratory of Plant Physiology, Institute of Horticulture, Lithuanian Research Centre for Agriculture and Forestry, Kauno St. 30, LT-54333 Babtai, Kaunas Dist., Lithuania; ausra.brazaityte@lammc.lt (A.B.); viktorija.vastakaite-kairiene@lammc.lt (V.V.-K.); asta.kupcinskiene@lammc.lt (A.K.); pavelas.duchovskis@lammc.lt (P.D.); giedre.samuoliene@lammc.lt (G.S.)

**Keywords:** inhibition, light-emitting diode, mycelium, pathogen, recovery

## Abstract

*Botrytis cinerea* is a ubiquitous necrotrophic pathogen causing grey mould in economically important crops. Light effect in horticulture is undeniable and fungi also react to light. Selected specific light-emitting diodes (LEDs) and photoperiods can be used for fungal pathogen inhibition. This study aimed to evaluate how LED light wavelengths and photoperiods affect the growth parameters of *B. cinerea*. The morphological (mycelium appearance, sclerotia distribution) and phenotypic (conidia presence and size, mycelium growth rate, recovery) characteristics of the fungal pathogen *B. cinerea* were evaluated under royal blue 455 nm, blue 470 nm, cyan 505 nm, yellow 590 nm, and red 627 nm LED lights at various photoperiods (4, 8, 12, 16, 20, 24 h). The results revealed that the light conditions and photoperiods influenced the *B. cinerea* morphological and phenotypic characteristics. Overall, the highest *B. cinerea* inhibition was under yellow (590 nm) LED light at 4 and 8 h photoperiods. Conidia did not form under blue 455 nm at 8, 16, 20, and 24 h photoperiods. Therefore, it can be assumed that the phenotypic and morphological features of *B. cinerea* depend on the specific photoperiod and LED light wavelength. The results allowed an exploration of original research approaches, raised new scientific questions for further investigation, and suggested new green plant protection solutions.

## 1. Introduction

*Botrytis cinerea* Pers.: Fr., which causes grey mould, is an important plant pathogen with a significant impact on a broad range of plants and yield rots as well as decreasing postharvest qualities [1,2,3,4]. *B. cinerea* is a necrotrophic fungal pathogen that infects more than 596 various plant genera. It infects plants, causing grey mould, and infects various plant parts such as the flowers, petioles, fruit, leaves, and stems and often starts early as blossom blight. Frequently, the pathogen is invisible until fruit softness during ripening. *B. cinerea,* as a broad host range pathogen, causes pre- and postharvest losses [1,5,6]. *Botrytis* spp. causes losses, depending on the pathogen development after harvest, through the entire postharvest chain [2]. The annual economic losses of vegetables and fresh fruit caused by *B. cinerea* range from USD 10 billion to USD 100 billion worldwide. *B. cinerea* is considered to be the second most important plant pathogen [3].

Food production contamination is a serious problem and essential to humans. Due to unsafe food, many people become sick. This problem originates from various sources such as phytotoxins, chemical pesticides, food processing chemicals, and others [7]. Chemical pesticide residues as contaminants are present in food production in higher than safe concentrations [8,9,10]. However, in agriculture, conventional chemical fungicides are used for disease control. Plant diseases are controlled by routine applications with intervals of 7–21 days (depending on the pesticide) from the leaf emergence until harvest [1,11]. In addition, chemical pesticides negatively affect the environment and beneficial organisms as well as polluting water, soil, and food and affect animal and human health. Furthermore, the overuse of chemicals induces pathogen resistance, which emerges due to frequent pesticide use [6,8,9,12,13].

The EU directive 2009/128/EC and Green Deal has led to the sustainable use of pesticides. Researchers all over the world are looking for more sustainable, innovative ways to control plant pathogens. Therefore, future plant protection should be based on the integrated control of harmful organisms whilst prioritising the least harmful methods to humans and the environment [14,15,16,17,18]. It has been reported that various physical, chemical, and biological (bio-fungicides, plant extracts, etc.) strategies have been applied to prolong and maintain the shelf-life of horticultural crops [14,15,16,18,19].

The role of visible light in agriculture and horticulture is undeniable, as light is a source of energy and induces photosynthesis, which is crucial for plant growth and development. Supplemental lighting may prolong the cropping season and leads to nutritional quality improvement, nitrate concentration decrease, yield quality increase, and other parameters of plants grown in a closed environment agriculture [20,21,22,23]. Light also influences fungi and it controls the physiological and morphological responses. Fungi can sense light using up to 11 photoreceptors. Additionally, light signalling could be linked with the metabolic pathway, sporulation, or secondary metabolites production [24]. *B. cinerea* also has photoreceptors and reacts to light. The asexual and sexual development of *B. cinerea* depend on light illumination [25]. Light affects mycelial growth, conidiation, and sclerotia formation [3,25]. Over traditional light-emitting diode (LED) use, the specific wavelengths are targeted to plant pathogen control [14,16,19]. A literature review shows that blue LED light could suppress the germination and sporulation of *Botrytis* spp., *Phomopsis* spp., *Aspergillus* spp., and *Penicillium* spp. and far-red, blue, and red inhibit *Aspergillus* spp. and other pathogens [12,13,26,27]. Red LED enhanced a resistance to *S. fuliginea* in cucumbers [28]. Red, blue, and green LED lights can induce a systemic resistance to fungal pathogens [14]. It has been reported that blue LED suppresses *B. cinerea,* and 50–150 μ mol m^−2^ s^−1^ blue LED induced *B. cinerea* resistance in tomatoes [12]. However, it raises the question about the intensity and duration (photoperiod) of the light. It has been reported that 12 h and 24 h blue LED exposure reduced *P. digitatum* [27]. A cycle of 12 h of blue LED followed by a 12 h dark period per day effectively reduced *P. digitatum* mycelium [29]. Blue (450–460 nm) and purple (400–410 nm) lights inhibited *B. cinerea* mycelium at a 12 h photoperiod [30]. 

Previous studies reported that LED light influences plants and pathogens. There are many reports on the effects of blue light but there is a lack of other light spectra. Additionally, there is a lack of a direct impact on *B. cinerea*. Therefore, we assumed that a specific LED light spectrum could suppress or stimulate plant pathogens. Not all studies evaluated the morphological and phenotypical characteristics of *B. cinerea*, which could be related to the ability of the pathogen to spread and infect plant tissues. Therefore, this study aims to evaluate how monochromatic LED light wavelengths and photoperiods affect the growth parameters of *B. cinerea* in vitro.

## 2. Materials and Methods

The research was conducted at the Lithuanian Research Centre for Agriculture and Forestry Institute of Horticulture (LAMMC IH) Laboratory of Plant Physiology under controlled environment conditions in 2018 and 2019. 

### 2.1. Fungal Isolate

The *B. cinerea* LT13B_FRA_76 isolate used in this study was from the LAMMC IH Laboratory of Plant Protection isolate collection. *B. cinerea* from rotten strawberry fruit was morphologically identified and verified by a species-specific PCR, according to [4]. The single-spore isolates were cultivated on potato dextrose agar (PDA) (Liofilchem, Roseto degli Abruzzi, Italy). 

### 2.2. Light Treatments

The light sources were five monochromatic LED light arrays: royal blue 455 nm (LXHL-LR3C), blue 470 nm (LXHL-LB3C), cyan 505 nm (LXHL-LE3C), yellow 590 nm (LXHL-LL3C), and red 627 nm (LXHL-LD3C) (Philips Lumileds Lighting Company, San Jose, CA, USA). The distance between the fungal samples and light sources was adjusted to the photosynthetic photon flux density (PPFD) of 20 ± 2 μ/mol m^−^^2^ s^−1^. The light intensity was measured by a photometer-radiometer RF-100 (Sonopan, Bialystok, Poland). 

### 2.3. B. cinerea Cultivation Parameters

The isolates were cultivated on PDA to investigate the morphological and phenotypic characteristics of *B. cinerea* under different LED lights and photoperiods. Isolate plugs of 7 mm diameter, mycelium side-down, were inoculated in the centre of Petri dishes. The inoculated Petri were assigned to different LED light wavelengths and 4, 8, 12, 16, 20, and 24 h photoperiods in closed, controlled environment growth chambers at 22 ± 2 °C for 7 days. The relative air humidity was 60–70%. The control plates with the pathogens were in complete darkness. There were four replicates per treatment. The experiment was repeated twice. 

### 2.4. Evaluation of the Morphological and Phenotypic Characteristics

*B. cinerea* was investigated for morphological (mycelium appearance, sclerotia distribution) and phenotypic (conidia presence and size, mycelium growth rate) characteristics.

The mycelium growth rates were determined by measuring the colony diameter (mm) of the isolates daily for seven days after inoculation (1–7 DAI), as described [31]. Four Petri plates were used per treatment (wavelength × photoperiod). The mycelium growth rates (mm day^−1^) were calculated as an average length and width increase per day. The mycelium growth rate was used to calculate the mycelial growth inhibition (MGI) [32], as follows:

$$\mathrm{MGI}\;\left(\%\right)=\frac{C-T}{C}\times 100$$

where C is the diameter of the pathogen mycelium growth rate in the control, mm, and T is the diameter of the pathogen mycelium growth rate in treatment, mm. 

The mycelial growth curve (AUMGC) was calculated [33]:

$$\mathrm{AUMGC}=\sum (y_{i}+y_{i}+1)/2\;*\;\mathrm{dti})/n$$

where *yi* = mean mycelium diameter in the four observations, mm, dti = the interval between the evaluations, and n = the evaluation period. 

The mycelium appearance, sclerotia distribution, conidia presence, and size (width and length) were evaluated after 7 DAI. Conidia were evaluated at the edge of the Petri. The evaluation of conidia was carried out on microscope slides with a Nikon Eclipse 80i microscope of 40 × magnification. Conidia were evaluated for presence, length (μm), and width (μm). A six-point scale was used to assess the mycelium appearance and sclerotia distribution: M1—mycelium without conidia; M2—mycelium with conidia; M3—mycelium masses; M4—thick mycelium; S0—no sclerotia; S1—sclerotia at the edge of the Petri; S2—arranged in circles; S3—large and irregular; and S4—small [34]. 

To evaluate the recovery after different LED light illuminations and photoperiods, the re-isolation was conducted after 7 DAI. The growth (mm) rate of the re-isolated fungi mycelium was measured after 48 h.

### 2.5. Statistical Analysis

SAS Enterprise Guide version 7.1 (SAS Inc., Cary, NC, USA) was used for the statistical analysis. An ANOVA and Duncan’s multiple range test (*p* < 0.05) were used to compare the obtained means. Conidia size and re-isolation data were expressed as mean ± standard deviation.

## 3. Results

**Figure 1:**
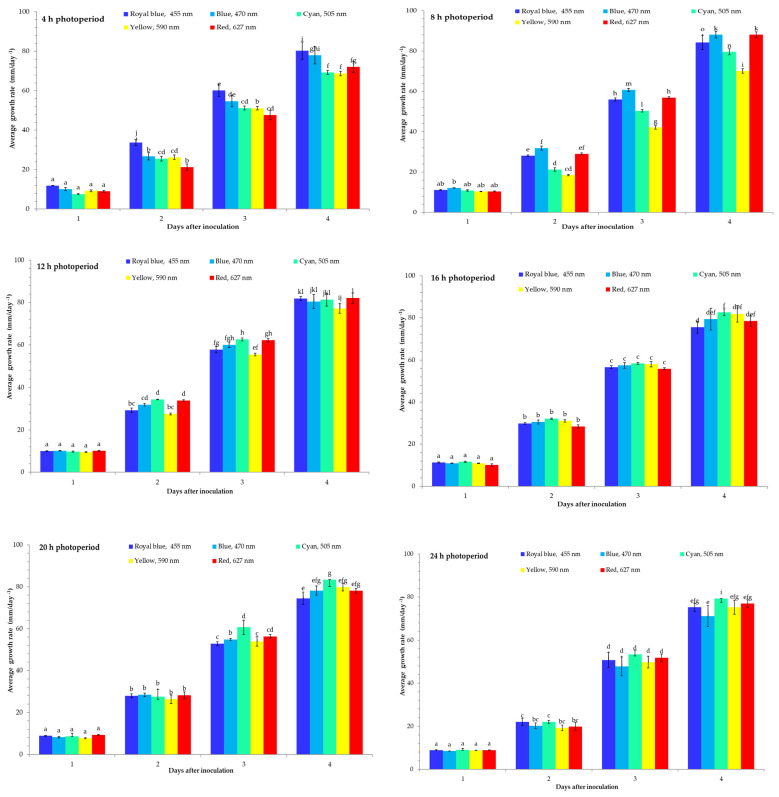
The average mycelium growth rate of illuminated *Botrytis cinerea* under 4, 8, 12, 16, 20, and 24 h photoperiods and different wavelengths. All values in the figure are expressed as mean ± standard error (n = 4). Means with different letters are significantly different at the *p* < 0.05 level according to Duncan’s multiple range test.

The *B. cinerea* isolates exposed to different LED light wavelengths and photoperiods showed differences in the morphological and phenotypic characteristics (Figure 1, Figure S1, Table 1, Table 2 and Table 3). The plugs on the plates were exposed to different LED light wavelengths at a 20 ± 2 μ/mol m^−^^2^ s^−1^ PPFD intensity. 

The light conditions influenced the *B. cinerea* mycelium growth rate and it was different for each wavelength (Figure 1, Figure S1). The results demonstrated that at a 4 h photoperiod, *B. cinerea* acted differently under different wavelengths. The lowest mycelium growth after 1 DAI was under a cyan (505 nm) LED light and after 2–3 DAI under a red (627 nm) LED light; however, at 4 DAI it was lowest under a yellow (590 nm) LED light compared with the other treatments. In addition, the highest *B. cinerea* mycelium growth rate was observed under a royal blue (455 nm) LED light after 1–4 DAI of incubation (Figure 1). The highest inhibition against the *B. cinerea* mycelium growth had a yellow (590 nm) LED light after 4 DAI. The MGI of the *B. cinerea* mycelium growth under illumination at different wavelengths reached only 6.3% with a yellow LED light after 4 DAI. The MGI inhibition of *B. cinerea* mycelium growth of the cyan (505 nm) LED light treatment was 5.5%.

The results showed that at an 8 h photoperiod, a blue (470 nm) LED light increased *B. cinerea* mycelium growth after 1–4 DAI. However, at an 8 h photoperiod, the *B. cinerea* mycelium diameter was the lowest under a yellow (590 nm) LED light after 1–4 DAI. The MGI of *B. cinerea* for the yellow (590 nm) LED light was 0%. The *B. cinerea* mycelium acted similarly at a 12 h photoperiod. The highest inhibition at a 12 h photoperiod against the *B. cinerea* mycelium growth was a yellow (590 nm) LED light at 1–4 DAI (Figure 1). However, the red (627 nm) LED light treatments increased the mycelium growth at 4 DAI. The lowest mycelium growth rate was under the red (627 nm) LED light after 1–3 DAI (Figure 1). However, at 4 DAI, the highest inhibition of *B. cinerea* mycelium growth had a royal blue (455 nm) LED light. Cyan (505 nm) increased the *B. cinerea* mycelium growth after 1–4 DAI. The MGI inhibition rate under royal blue reached only 5.3% and red 1.6%. Under a 20 h photoperiod, the radial growth of *B. cinerea* was similar to that under 16 h. The mycelium growth rate was lowest under yellow at 1–2 DAI. However, the highest inhibition of mycelium growth was observed under royal blue (455 nm) at a 20 h photoperiod at 3–4 DAI. The highest mycelium growth was observed under cyan (505 nm) at 4 DAI. The MGI inhibition rate due to the royal blue (455 nm) LED light treatment was low at 5.5%. However, the fungus acted differently at a 24 h photoperiod (Figure 1). The highest inhibition of the *B. cinerea* mycelium growth rate was observed in the blue (470 nm) LED light at 1–4 DAI. The highest mycelium growth was observed in the cyan (505 nm) LED light at 1–4 DAI. However, the MGI inhibition rate due to the blue (470 nm) LED light treatment was low.

Various wavelengths slightly reduced the mycelial growth curve (AUMGC) of the *B. cinerea* at different photoperiods (Table 1). For example, *B. cinerea* at the 4 h photoperiod under the red (627 nm) LED light reduced the AUMGC by 18.9%; at an 8 h photoperiod under the yellow (590 nm) LED light, the AUMGC was 17.8%. Additionally, at a 12 h photoperiod, *B. cinerea* reduced the AUMGC by 21.3% under the yellow (590 nm) LED light. However, under the 16 h photoperiod, *B. cinerea* reduced the AUMGC under the red (627 nm) LED light by 21.7%. *B. cinerea* was reduced by the royal blue (455 nm) LED light by 20.6% of the AUMGC compared with the other wavelengths. *B. cinerea* under the blue (470 nm) LED light was reduced by 18.6% of the AUMGC.

*B. cinerea* demonstrated a conidia size variation under the various LED light wavelengths and photoperiods (Table 2). The conidial dimensions differed among the isolates under the different photoperiods, ranging from 6.4 to 7.9 μm in width and 8.3 to 12.2 μm in length. The results revealed that the *B. cinerea* conidia width at the 4 h photoperiod was the smallest under the cyan (505 nm) LED light and the largest under the red (627 nm) LED light. However, the length of the conidia was smallest under the royal blue (455 nm) LED light and the largest under the red (627 nm) LED light at a 4 h photoperiod. Additionally, under the 8 h photoperiod, conidia were absent under the royal blue (455 nm). However, the largest width was under the blue (470 nm) and length was under the cyan (505 nm) LED light. At a 12 h photoperiod, conidia were absent under the blue (470 nm) LED light. The diameter of the royal blue (455 nm) conidia was the largest at 7.9 μm in width and under the red (627 nm) at 12.2 μm in length. However, at a 16 h photoperiod, the width of the conidia was absent under the royal blue (455 nm) and blue (470 nm) LED lights. The highest conidia width and length at a 16 h photoperiod was with the yellow (590 nm) LED light at 6.4 × 10.2 μm. Conidia were absent at the 20 h photoperiod under the royal blue (455 nm) and cyan (505 nm) LED lights. The highest width and length were at the red (627 nm) LED light of 7.3 × 11.2 μm. Conidia at the 24 h photoperiod was absent under the royal blue (455 nm), blue (470 nm) and cyan (505 nm) LED lights. The conidia dimension was the largest in width under the yellow (590 nm) (7.3 μm) and red (627 nm) LED lights in length (9.4 μm) at a 24 h photoperiod.

The morphological distribution among the different wavelengths and photoperiods was evaluated after 7 DAI. *B. cinerea* was categorised into groups by the mycelium appearance and sclerotia distribution under the different photoperiods and LED light wavelengths (Table 3). The *B. cinerea* isolates were grouped according to the mycelium appearance and sclerotia distribution under the light treatments and photoperiods.

Two groups of mycelia were defined based on their mycelium appearance and five groups of sclerotia distribution. The mycelium appearance varied under all photoperiods. The mycelium varied from mycelium without conidia (M1) to mycelium with conidia (M2). The mycelium appearance at the 4 h photoperiod under all monochromatic LED lights was identified with conidia (M2). 

The sclerotia distribution also varied among the different photoperiods and LED light wavelengths. The sclerotia varied from no sclerotia, sclerotia in the edge of the Petri, arranged in circles, or large irregular and small sclerotia. The results demonstrated that a 4 h photoperiod influenced the sclerotia differences. The sclerotia under royal blue (455 nm) were in the edge of the Petri (S1), blue (470 nm) and yellow (590 nm) were large and irregular (S3), red (627 nm) was arranged in circles (S2), and there were no sclerotia (S0) under cyan (505 nm). The 8 h photoperiod gave a lower variation under the different wavelengths. The sclerotia under royal blue (455 nm) were arranged in circles (S2); however, blue (470 nm) and red (627 nm) were at the edge of the Petri (S1). Under cyan (505 nm) and yellow (590 nm), the sclerotia were large and irregular (S3) at the 8 h photoperiod. 

Additionally, the sclerotia distribution under the 12 h photoperiod varied between the wavelengths. The obtained results of the sclerotia distribution showed that royal blue (455 nm) and cyan (505 nm) were large and irregular (S3) and yellow (590 nm) and red (627 nm) LED lights were small (S4); however, under the blue (470 nm) LED light, there were no sclerotia (S0). The 16 h photoperiod also influenced the sclerotia differences. The sclerotia under the royal blue (455 nm) LED light was at the edge of the Petri (S1). Under cyan (505 nm), yellow (590 nm), and red (627 nm) LED light, the sclerotia were large and irregular (S3). Under the blue (470 nm) LED light there were no sclerotia (S0). The results showed that there were no sclerotia (S0) under all wavelengths at the 20 h photoperiod. The sclerotia under the royal blue (455 nm) LED light were arranged in circles (S2) at the 24 h photoperiod. However, under the blue (470 nm) and red (627 nm) LED lights, there were no sclerotia (S0); cyan (505 nm) and yellow (590 nm) were at the edge of the Petri (S1). 

The re-isolation was performed after 7 DAI to determine the recovery of *B. cinerea* after illumination under different wavelengths (Table 4). The obtained data indicated that at the 4 h photoperiod under blue, the recovery was slower compared with the other wavelengths. *B. cinerea* under royal blue recovered faster than other LED lights. However, at the 8 h photoperiod, the slowest recovery was observed under the red (627 nm) and faster under the royal blue (455 nm) LED light. At a 12 h photoperiod under a blue (470 nm) and a red (627 nm) LED light, the recovery was slower compared with others.

Moreover, under a royal blue (455 nm) LED light, *B. cinerea* recovered faster at a 12 h photoperiod. At a 16 h photoperiod under a royal blue (455 nm) LED light, the fungus recovered and under a yellow (590 nm) LED light, recovery was slower than under other wavelengths. However, at a 20 h photoperiod under a yellow (590 nm) LED light, *B. cinerea* recovered faster; under the blue (470 nm) LED light, it was slower. The results showed that at a 24 h photoperiod, the recovery was opposite to the 20 h photoperiod. The slowest recovery was under the yellow (590 nm) LED light and fastest under the blue (470 nm) LED light. 

## 4. Discussion

An LED light could serve as an environmentally friendly tool in controlled environment conditions. LED light technology allows the selection of a specific light spectrum and photoperiod to prevent food crop damage by pathogens. The inhibitory effect of different LED light wavelengths and photoperiods on the morphological and phenotypic characteristics of the fungus could be related to the ability of the pathogen to spread and infect plant tissues. Therefore, it was substantial to evaluate the LED light and photoperiod effects on *B. cinerea*. We evaluated how different LED light wavelengths and photoperiods affected the phenotypic and morphological features of *B. cinerea*. 

Literature research has shown only an effect on only one specific photoperiod or wavelength but not more complex than our research [27,30,35,36]. There is a theory that fungi may use light combined with the circadian clock to adapt to stress and produce reproductive structures at the right time and place [24]. In this study, we observed that *B. cinerea* acted differently under different photoperiods and LED light wavelengths. 

Recently, light effects on fungi growth, development, inhibition, and reduction have been reported. For example, the impact of light quality rose *Podosphaera pannosa* growth and development [36]. On the other hand, a light–dark regime or a continuous blue light (40 μmol·m^−^^2^·s^−^^1^) reduced the mycelium growth of *P. italicum* and *P. citri* [27]. Our results showed that *B. cinerea* mycelium grew under different photoperiods and wavelengths; however, there were differences in the morphological and phenotypic characteristics. Certain wavelengths and photoperiods can inhibit or increase mycelium growth. 

Overall, our data showed that the highest *B. cinerea* inhibition was under a yellow (590 nm) LED light at a 4 h photoperiod. The 8 and 12 h photoperiods also showed a high inhibition under a yellow (590 nm) LED light at 4 DAI. The 24 h photoperiod inhibited *B. cinerea* mycelium growth under a blue (470 nm) LED light at 3–4 DAI. However, the mycelium growth was increased under a royal blue (455 nm) LED light at a 4 h photoperiod and a blue (470 nm) light at an 8 h photoperiod after 4 DAI. Additionally, the mycelium growth increased under the blue (470 nm) and red (627 nm) LED lights at a 12 h photoperiod. The mycelium growth increased under cyan (505 nm) at 16, 20, and 24 h photoperiods. It has been reported that a 12 h photoperiod illumination of purple light (400–410 nm) and blue (450–460 nm) significantly inhibited *B. cinerea* mycelium growth [30]. In our research under a royal blue (455 nm) LED light at 4, 8, 12, and 16 h photoperiods, *B. cinerea* recovered faster. Our results showed that LED light also influenced conidia formation. The results showed that under royal blue (455 nm) at 8, 16, 20, and 24 h photoperiods, the sporulation was suppressed, and conidia did not form. Additionally, conidia did not form under blue (470 nm) at 12, 16, and 24 h photoperiods. Blue (405 nm) light under a 12 h photoperiod inhibited *B. cinerea* spores on detached tomato leaves [37]. Additionally, blue (420–520 nm, peak 465 nm) light reduced *Podosphaera pannosa* conidia germination [35]. It was reported that blue (410–540 nm) light inhibited conidia production of *P.* *digitatum* [27]. A blue (458 nm) LED light after 6 h influenced *Colletotrichum acutatum* conidial germination and germ tube growth [38]. A 12 h (followed by 12 h of a dark period) treatment per day effectively reduced *P. digitatum* mycelium [29]. It is known that light influences *B. cinerea* asexual reproduction. Blue light negatively affects conidia production as it inhibits the formation of conidiophores [39]. The absence or reduction of conidia production indicates that it is possible to prevent the fungal spread and postharvest contamination. In our research, the mycelium morphological type varied at all photoperiods. The mycelium appearance was without conidia (M1) to mycelium with conidia (M2). *B. cinerea* pathosystems developed a less fluffy mould than in dark-treated samples although red, white, and dark conditions caused similar soft decay [40]. Our data indicated that *B. cinerea* sclerotia distribution and mycelium appearance differed under different photoperiods and LED light wavelengths. The sclerotia varied from no sclerotia, sclerotia at the edge of the Petri, arranged in circles, or large irregular and small sclerotia. Our results also suggested that the phenotypic and morphological features of *B. cinerea* depended on the specific photoperiod and LED light wavelength. Therefore, we could hypothesise that the illumination effect could be short-term and adapting the illumination of a fungus with different wavelengths should be in cycles to suspend the pathogen recovery. 

## 5. Conclusions

The research of this paper reflects global trends, relevant pathogen controls, and management methods based on food safety and sustainable agriculture principles. The results allowed the exploration of original research approaches and suggested new green plant protection solutions to meet world food contamination problems as well as raised new scientific questions for further investigations to develop innovative plant protection methods of *B. cinerea* management. Developing an optimal illumination regime before implementing an LED light treatment to control against *B. cinerea* is essential. However, illumination by LED lights for fungal pathogens should be combined with other disease control methods. This research is at an initial stage and further research is required to develop alternative plant protection methods of strawberry *B. cinerea* management.

## Figures and Tables

**Table 1:** The mycelial growth curve (AUMGC) of *Botrytis cinerea* under different wavelengths and photoperiods.

LED Light	Photoperiods					
	4 h	8 h	12 h	16 h	20 h	24 h
Royal Blue, 455 nm	23.4 ± 1.3	22.6 ± 0.7	22.5 ± 0.6	21.8 ± 0.6	20.6 ± 0.8	19.7 ± 1.0
Blue, 470 nm	21.3 ± 1.4	24.2 ± 0.6	22.9 ± 0.8	22.4 ± 1.1	21.3 ± 0.6	18.6 ± 1.5
Cyan, 505 nm	19.3 ± 0.6	20.4 ± 0.5	23.6 ± 0.7	23.2 ± 0.5	22.7 ± 1.2	20.6 ± 0.5
Yellow, 590 nm	19.5 ± 0.6	17.8 ± 0.4	21.3 ± 0.6	22.9 ± 0.9	21.1 ± 0.9	19.3 ± 1.1
Red, 627 nm	18.9 ± 1.0	23.2 ± 0.5	23.7 ± 0.6	21.7 ± 0.7	21.6 ± 0.6	19.8 ± 0.8

Data are expressed as mean ± standard deviation.

**Table 2:** Comparison of the average conidia size of *Botrytis cinerea* after illumination by various LED light wavelengths and photoperiods.

Conidia Size, μm	LED Light				
	Royal Blue,	Blue,	Cyan,	Yellow,	Red,
	455 nm	470 nm	505 nm	590 nm	627 nm
	4 h photoperiod				
Width	6.5 ± 0.3	6.6 ± 0.3	6.4 ± 0.3	6.6 ± 0.3	6.8 ± 0.3
Length	8.3 ± 0.2	8.9 ± 0.2	9.0 ± 0.6	8.5 ± 0.2	9.8 ± 0.3
	8 h photoperiod				
Width	0 ± 0	6.8 ± 0.2	6.5 ± 0.3	5.9 ± 0.5	6.6 ± 0.2
Length	0 ± 0	8.8 ± 0.1	8.9 ± 0.3	8.5 ± 0.4	8.8 ± 0.4
	12 h photoperiod				
Width	7.9 ± 0.2	0 ± 0	7.0 ± 0.0	7.1 ± 0.3	7.5 ± 0.4
Length	11.1 ± 0	0 ± 0	10.4 ± 0.5	9.9 ± 0.4	12.2 ± 0.6
	16 h photoperiod				
Width	0 ± 0	0 ± 0	6.2 ± 0.5	6.4 ± 0.2	6.2 ± 0.3
Length	0 ± 0	0 ± 0	8.9 ± 0.3	10.2 ± 0.1	9.5 ± 0.2
	20 h photoperiod				
Width	0 ± 0	5.7 ± 0.4	0 ± 0	6.6 ± 0.4	6.7 ± 0.3
Length	0 ± 0	8.6 ± 0.2	0 ± 0	10.4 ± 0.3	11.2 ± 0.2
	24 h photoperiod				
Width	0 ± 0	0 ± 0	0 ± 0	7.3 ± 0.2	6.3 ± 0.3
Length	0 ± 0	0 ± 0	0 ± 0	9.1 ± 0.4	9.4 ± 0.4

Data are expressed as mean ± standard deviation.

**Table 3:** The phenotypic classification of *Botrytis cinerea* after illumination by various LED light wavelengths and photoperiods.

	LED Light				
	Royal Blue, 455 nm	Blue, 470 nm	Cyan, 505 nm	Yellow, 590 nm	Red, 627 nm
	4 h photoperiod				
Sclerotia	S1	S3	S0	S3	S2
Mycelium	M2	M2	M2	M2	M2
	8 h photoperiod				
Sclerotia	S2	S1	S3	S3	S1
Mycelium	M1	M2	M2	M2	M2
	12 h photoperiod				
Sclerotia	S3	S0	S3	S4	S4
Mycelium	M2	M1	M2	M2	M2
	16 h photoperiod				
Sclerotia	S1	S0	S3	S3	S3
Mycelium	M1	M1	M2	M2	M2
	20 h photoperiod				
Sclerotia	S0	S0	S0	S0	S0
Mycelium	M1	M2	M1	M2	M2
	24 h photoperiod				
Sclerotia	S2	S0	S1	S1	S0
Mycelium	M1	M1	M1	M2	M2

M1: mycelium without conidia; M2: mycelium with conidia; S0: no sclerotia; S1: sclerotia at the edge of the Petri; S2: arranged in circles; S3: large and irregular; S4: small.

**Table 4:** The average recovery of the *Botrytis cinerea* growth rate after illumination by various LED light wavelengths and photoperiods.

LED Light				
Royal Blue,	Blue,	Cyan,	Yellow,	Red,
455 nm	470 nm	505 nm	590 nm	627 nm
4 h photoperiod				
59.8 ± 0.0	43.8 ± 0.3	59.0 ± 0.0	48.2 ± 3.3	47.1 ± 7.5
8 h photoperiod				
55.0 ± 1.0	44.8 ± 0.2	44.5 ± 2.0	43.8 ± 1.3	41.5 ± 3.5
12 h photoperiod				
51.5 ± 1.0	46.8 ± 2.8	50.0 ± 5.0	47.3 ± 1.8	46.8 ± 1.8
16 h photoperiod				
66.5 ± 5.7	51.5 ± 1.8	45.0 ± 5.5	60.5 ± 5.6	60.0 ± 1.0
20 h photoperiod				
71.0 ± 3.5	58.3 ± 4.7	70.5 ± 1.8	75.0 ± 8.5	64.0 ± 1.0
24 h photoperiod				
72.8 ± 0.7	84.0 ± 0.2	67.5 ± 4.0	65.8 ± 1.8	66.0 ± 1.3

Data are expressed as mean ± standard deviation.

## Data Availability

Please refer to suggested Data Availability Statements in section “MDPI Research Data Policies” at https://www.mdpi.com/ethics, accessed on 14 November 2021.

## Supplementary Materials

The following are available online at https://www.mdpi.com/article/10.3390/jof7110970/s1. Figure S1. The average *Botrytis cinerea* mycelium growth rate under different wavelengths and 4, 8, 12, 16, 20, and 24 h photoperiods. All values in the figure are expressed as mean ± standard error (n = 4). Means with different letters are significantly different at the *p* < 0.05 level according to Duncan’s multiple range test.
