# Supplementary material for: The Effect of Monochromatic LED Light Wavelengths and Photoperiods on Botrytis cinerea
Source: J Fungi (Basel). 2021 Nov 16;7(11):970. doi: 10.3390/jof7110970 (PMC8622904; doi:10.3390/jof7110970)
Supplement: Supplementary file 1 [file jof-07-00970-s001.zip › jof-1463418-supplementary.pdf]

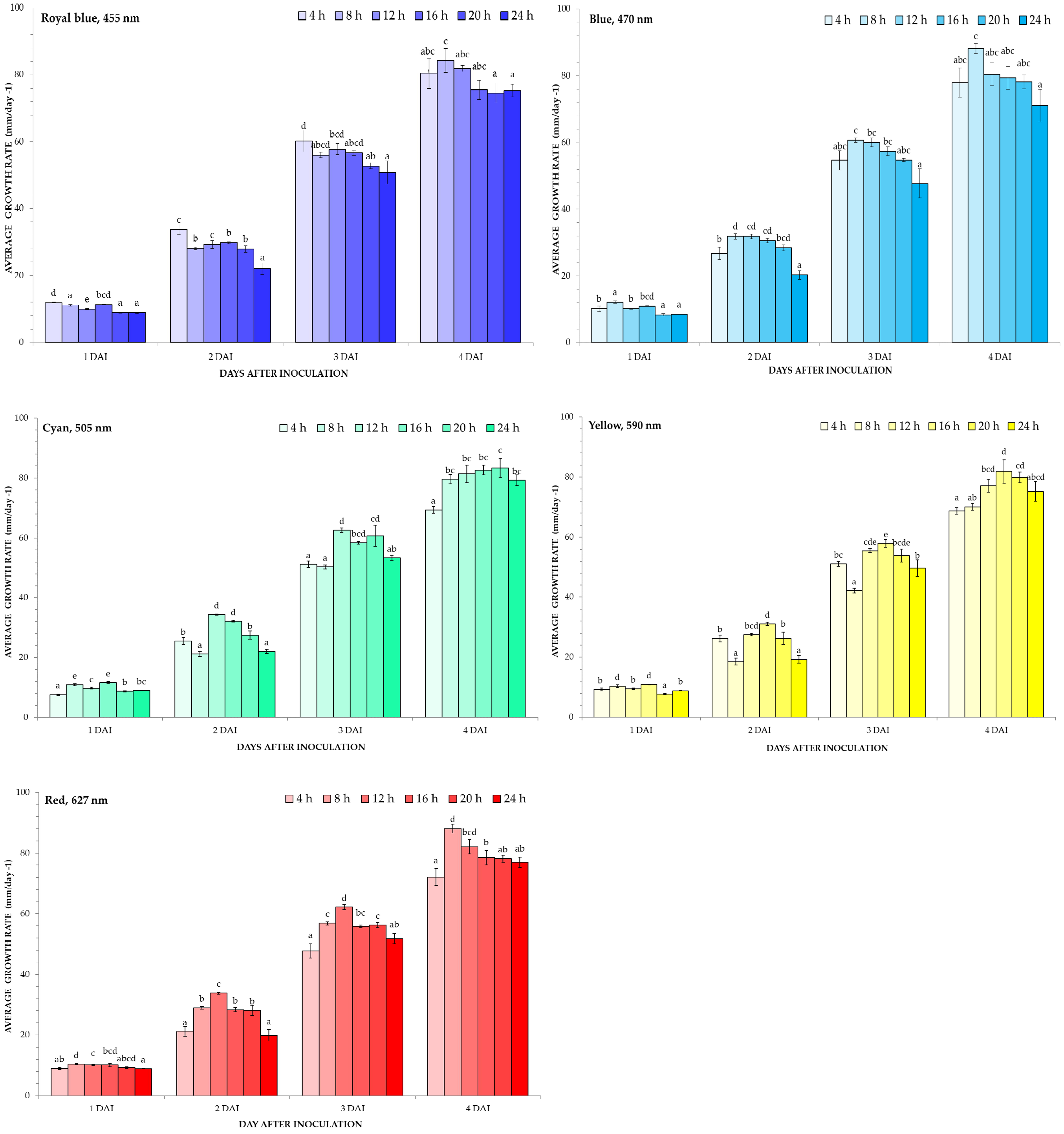

Figure S1. The average *Botrytis cinerea* mycelium growth rate under different wavelengths and 4, 8, 12, 16, 20, and 24 h photoperiods. All values in the figure are expressed as mean ± standard error (n = 4). Means with different letters are significantly different at the $p < 0.05$ level according to Duncan's multiple range test.
